# A Novel Missense Mutation of GATA4 in a Chinese Family with Congenital Heart Disease

**DOI:** 10.1371/journal.pone.0158904

**Published:** 2016-07-08

**Authors:** Xiaoqing Zhang, Jian Wang, Bo Wang, Sun Chen, Qihua Fu, Kun Sun

**Affiliations:** 1 Department of Laboratory Medicine, Shanghai Children’s Medical Center, Shanghai Jiao Tong University School of Medicine, Shanghai, China; 2 Department of Pediatric Cardiology, Xinhua Hospital, Shanghai Jiao Tong University School of Medicine, Shanghai, China; Tsinghua University, CHINA

## Abstract

**Background:**

Congenital heart disease (CHD) is the most prevalent type of birth defect in human, with high morbidity in infant. Several genes essential for heart development have been identified. GATA4 is a pivotal transcription factor that can regulate the cardiac development. Many GATA4 mutations have been identified in patients with different types of CHD.

**Aims:**

In this study, the NKX2-5, HAND1 and GATA4 coding regions were sequenced in a family spanning three generations in which seven patients had CHD. Disease-causing potential variation in this family was evaluated by bioinformatics programs and the transcriptional activity of mutant protein was analyzed by the dual luciferase reporter assay.

**Results:**

A novel GATA4 mutation, c.C931T (p.R311W), was identified and co-segregated with the affected patients in this family. The bioinformatics programs predicted this heterozygous mutation to be deleterious and the cross-species alignment of GATA4 sequences showed that the mutation occurred within a highly conserved amino acid. Even though it resided in the nuclear localization signal domain, the mutant protein didn’t alter its intracellular distribution. Nevertheless, further luciferase reporter assay demonstrated that the p.R311W mutation reduced the ability of GATA4 to activate its downstream target gene.

**Conclusions:**

Our study identified a novel mutation in GATA4 that likely contributed to the CHD in this family. This finding expanded the spectrum of GATA4 mutations and underscored the pathogenic correlation between GATA4 mutations and CHD.

## Introduction

Congenital heart disease (CHD) is the most common developmental abnormality, with a postnatal incidence of 1% [1]. During the development of heart, multiple molecular pathways are involved. Emerging evidences have indicated the crucial role of genetic defects in the occurrence of CHD. Pathogenic gene mutations, chromosomal aberrations and microRNA lesion can all result in CHD [2]. Tetralogy of Fallot (TOF) is the most common type of cyanotic CHD [3]. Several transcription factors essential for cardiac development have been identified in causing TOF, including NKX2-5, HAND1 and GATA4 [4–7].

GATA4 belongs to a family of DNA binding proteins. They share highly conserved zinc finger domains and target the consensus DNA sequence “GATA” element [8]. In vertebrates, six members of the GATA family have been identified. GATA1, GATA2 and GATA3 are mainly expressed in the hematopoietic cells, whereas GATA4, GATA5 and GATA6 are predominantly expressed in tissues such as the heart, liver and gonads [9]. During the development, GATA4 is highly expressed in the embryonic heart and adult cardiomyocytes. By cooperating with target genes, GATA4 can regulate multiple processes essential for normal cardiac morphogenesis [10].

Small changes of GATA4 can dramatically influence cardiac development and embryonic survival. In mice, GATA4 homozygous knockout embryos die in early embryonic stage because of failed heart tube formation and abnormal ventral folding [11]. Mouse embryos expressing reduced GATA4 protein display congenital cardiac defects including common atrioventricular canal, double outlets of the right ventricle and myocardial hypoplasia [12]. In Xenopus embryos, GATA4 is sufficient to induce functional cardiomyocyte differentiation in embryonic ectoderm [13]. Functional experiments in chick, fly and fish also demonstrate the importance of GATA4 in their heart development [14]. In human, GATA4 mutations can lead to many kinds of congenital heart diseases [15]. Till now, more than 90 missense mutations have been reported in CHD patients (S1 Table).

In our study, a novel heterozygous GATA4 mutation, c.C931T (p.R311W), was identified in a family spanning three generations and seven individuals in this family had CHD. All family members were negative for mutation screening in NKX2-5 and HAND1. The proband of this family was affected with TOF. Detailed clinical and genetic evaluations were reviewed for all family members, and displayed an autosomal dominant pattern of inheritance. The p.R311W mutation was functionally analyzed, and in vitro assays showed that the mutation compromised the ability of GATA4 to transactivate its downstream target gene. This finding expanded the spectrum of GATA4 mutations and supported the essential role of GATA4 in cardiac development.

## Materials and Methods

### Study Subjects

**Fig 1:**
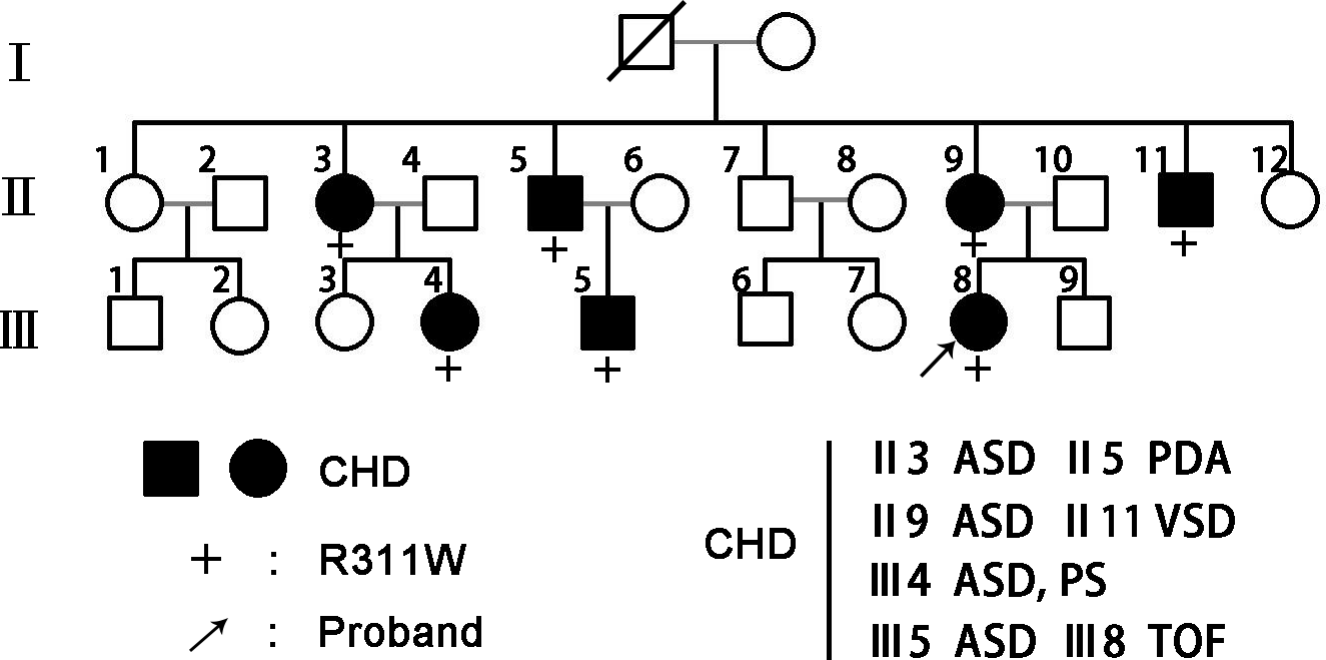
GATA4 mutation segregates with familial CHD. Family members in the pedigree chart are indicated by generations (Ⅰ-Ⅲ) and numbers. Males are symbolized by squares while females are circles and the symbols with a slash indicate the deceased members. Patients affected with CHD are designated with filled symbols and the plus signs (+) represent carriers of the heterozygous R311W mutation. The proband of the family is indicated by arrow.

A three-generation pedigree with congenital heart disease from Guizhou Province, China was enrolled in the study. All family members were clinically evaluated by reviewing patient history, physical examinations and medical records. Seven patients were diagnosed to have congenital heart disease (II-3, II-5, II-9, II-11, III-4, III-5, and III-8; Fig 1). The proband of this family was a girl aged 7 years (Patient III-8). Her medical history reported that she was referred for evaluation of a murmur just after she was born and was diagnosed by echocardiograms to have TOF at 4 years old. None of the 7 affected members had any malformations in other organs, showing that it was a familial nonsyndromic CHD with a pattern of autosomal dominant inheritance. Written consent was obtained from all participants. For children enrolled in our study, a signed informed consent was obtained from their guardians. This research was approved by the Ethics Committee of Shanghai Children's Medical Center.

### Genetic studies

Peripheral blood samples of all cases were obtained and genomic DNA was extracted using QIAmp DNA Blood Mini Kit (QIAGEN, Hilden, Germany) by standard protocols. DNA was quality tested by optical density (OD) 260/280 nm ratios, quantified by UV spectrophotometry (NanoDrop Technologies, Wilmington, DE, USA), and stored at -20°C until use. All exon regions and flanking intronic sequences of NKX2-5, HAND1and GATA4 were amplified by polymerase chain reaction (PCR) to screen gene mutations.

### Multiple sequence alignments of GATA4

To evaluate the evolutionary conservation of the mutation, protein sequences of GATA4 from 7 animal species including human (Homo sapiens: NP_001295022), pig (Sus scrofa: NP_999458), cattle (Bos taurus: NP_001179806), rat (Rattus norvegicus: NP_653331), mouse (Mus musculus: NP_001297539), rabbit (Oryctolagus cuniculus: XP_008247110), dog (Canis lupus: NP_001041577) were aligned using the BioEdit program.

### Disease-causing potential prediction of the GATA4 mutation

To predict the disease-causing potential of GATA4 mutation in our report, several bioinformatic programs as MutationTaster (http://www.mutationtaster.org), PolyPhen-2 (http://genetics.bwh.harvard.edu/pph2) and SIFT (http://sift.jcvi.org/) were used. Swiss PdbViewer software version 3.7 was applied to analyze the three-dimensional conformation of GATA4. We selected the neighboring residues of amino 311 for H-bond computation to assess the influence of p.R311W mutation.

### Plasmid Constructs and Site Directed Mutagenesis

Human GATA4 cDNA (ENST00000532059) was PCR amplified and cloned into pcDNA3.1 (+) expression vector. The identified mutation was introduced using the QuikChange II Site-Directed Mutagenesis Kit (Stratagene, La Jolla, CA) following manufacturer’s instructions. Both wild-type and mutant inserts were sequenced in sense and antisense directions to confirm the desired sequence and exclude any further base exchanges.

### Cell Culture and Transfection

Hela cell line was purchased from the Type Culture Collection of the Chinese Academy of Sciences (Shanghai, China) and cultured in the DMEM medium supplemented with 10% FBS, penicillin (100 units/ml) and streptomycin (100 μg/ml). Cells were incubated at 37°C in a humid atmosphere with 5% CO_2_, 95% air. Plasmids were transfected into Hela cells with Lipofectamine 3000 reagent (Invitrogen, Carlsbad, CA) using the methodology recommended by the manufacturer.

### Western Blot and Immunofluorescence

For overexpression experiments, Hela cells were transfected with pcDNA3.1-GATA4-wt or mutant plasmid DNA in 24-well plates. To confirm that GATA4 protein was expressed in transiently transfected cells, western blot analysis was carried out. Cells were harvested 48hr after transfection and lysed in 1×SDS buffer. Cell lysates were analyzed by sodium dodecyl sulfate-polyacrylamide gel electrophoresis and transferred onto PVDF membranes (Amersham Biosciences, Piscataway, USA). Blocking of the membrane was done with 5% BSA in Tris-buffered saline containing 0.1% Tween-20. Then we incubated the membrane with anti-human GATA4 antibody (Bioworld, Nanjing, China) and anti-GAPDH antibody (Bioworld) at a dilution of 1:1000. The antigen-antibody complex was then incubated with horseradish peroxidase-conjugated secondary antibodies (Bioworld) and visualized with an ImageQuant LAS 4000 (GE Healthcare life science, USA). For immunofluorescence, Hela cells which have been transfected with GATA4 wide-type or mutant plasmid were fixed using 4% paraformaldehyde/PBS for 20min, permeabilized with 1% Triton X-100/PBS for 10min, and blocked using 1% BSA/PBS solution for 30min. Treated cells were incubated with anti-human GATA4 antibody at a dilution of 1:1000 and then with fluorescein isothiocyanate conjugated secondary antibody for 1hr. The cells were observed using a Laser Scanning Confocal Microscope (Leica, Germany).

### Luciferase Assay

The human ANF promoter from -644 to +35 was PCR amplified and inserted into the pGL4.10 vector (Promega, Madison, USA) to produce ANF-Luc [4]. To analyze the transcriptional activity of GATA4 protein, Hela cells were transfected with the human ANF-Luc plasmid, GATA4-pcDNA3.1 plasmid (wide-type, R311W mutant) as well as an internal control reporter plasmid pRL-TK (Promega). Briefly, ∼1.0 × 10^5^ cells were seeded per well in 24-well plates and incubated at 37°C in a CO_2_ incubator. At ∼80% confluence, the cells were cotransfected with 200 ng of pGL4.10-ANF luciferase reporter, 200 ng of pcDNA3.1-GATA4 plasmids (wild-type or R311W mutant) and 5 ng of pRL-TK. Thirty six hours after transfection, the luciferase activity of cells was measured with Dual-Glo luciferase assay system (Promega) according to the manufacturer’s instructions. Triple transfection experiments were performed three times for wild-type and GATA4 mutant. The student’s t-test was used for statistical analysis, and a P-value<0.05 was considered to be statistically significant.

## Results

### Identification of GATA4 mutation

**Fig 2:**
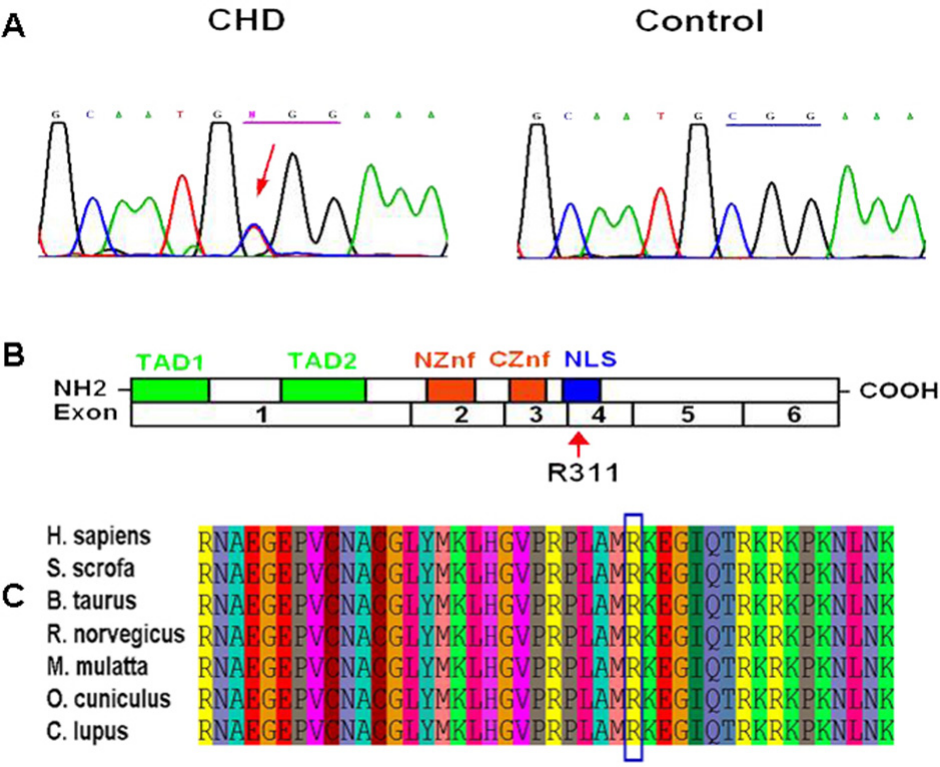
Distribution of the identified GATA4 mutation and multiple sequence alignment of GATA4 protein. (A) Sequencing results of the GATA4 mutation. The arrow indicates the heterozygous nucleotides of C/T. (B) A schematic diagram of GATA4 shows the location of the p.R311W mutation. (C) Multiple sequence alignment of the GATA4 indicates that residue 311 is highly evolutionarily conserved in mammals.

Sequencing of GATA4 revealed a heterozygous missense mutation c.C931T (p.R311W) in the proband. Then we tested the genomic DNA from other family members and found that the same mutation was present in all affected family members, but absent in unaffected individuals (Fig 1). In addition, we screened the NKX2-5 and HAND1 genes in this family; no mutations were identified. The multiple sequence alignments of GATA4 protein showed that the mutation occurred within a highly conserved amino acid, as presented in Fig 2, suggesting its critical function.

### Disease-causing potential of the GATA4 mutation

**Fig 3:**
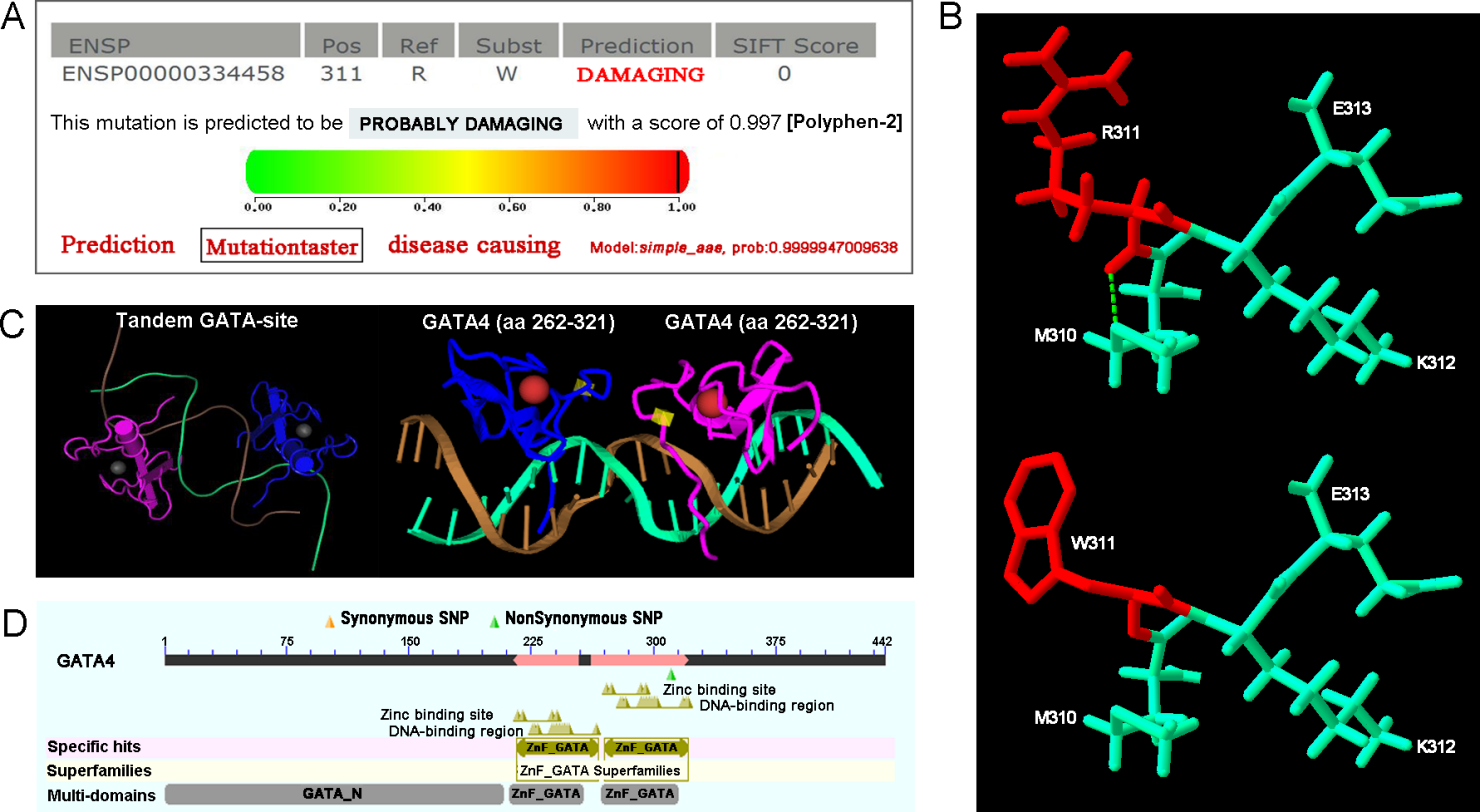
Disease-causing potential of the GATA4 mutation. (A) Functional significance of mutation prediction done by SIFT, PolyPhen-2 and MutationTaster. (B) Three-dimensional structure of GATA4 protein predicted by Swiss-PdbViewer. Upper panel is the reference structure; the lower panel is with arginine in position 311 mutated to tryptophan. (C) Double GATA4 bound to a tandem GATA4 binding site. Amino acid in position 311 is highlighted as yellow cube. (D) A schematic diagram of GATA4 protein.

The c.C931T mutation of GATA4 was predicted by SIFT to be damaging with a SIFT score of 0 which suggested a high ‘security’ of the prediction. Meanwhile, the pathogenicity of the mutation was also supported by Polyphen-2 and Mutationtaster with scores of nearly 1, predicting the mutation to be probably damaging and disease-causing, respectively (Fig 3A). High pathogenicity scores calculated by different prediction software suggested the deleterious effect of the mutation.

To further explore the functional effect of p.R311W, we used Swiss PdbViewer to predict the three-dimensional structure of GATA4 protein. Results showed that Arg311 was inclined to form multiple hydrogen bonding (H-bonds) and could make direct H-bond with Met310 (Fig 3B). Meanwhile, arginine was polar and positively charged which was ideal for binding negatively charged groups, e.g., nucleic acids. A model of GATA4-DNA binding domain was predicted by using the temple of highly conserved zinc finger domains of GATA3 (PDB code 3DFX with 80% sequence identity, Fig 3C). As shown in Fig 3C and 3D, p.R311 located in the DNA-binding domain. When arginine was mutated to the nonpolar hydrophobic amino acid tryptophan, the charge of the residue altered and might reduce GATA4 protein-DNA binding activity.

### Nuclear localization of GATA4 mutant protein

**Fig 4:**
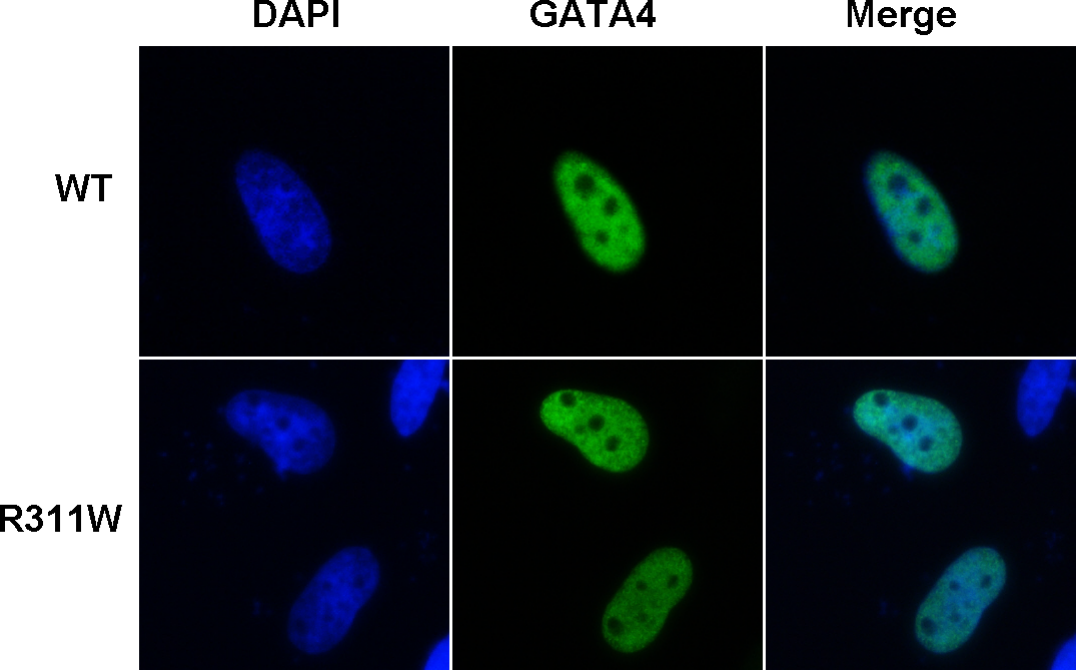
Normal subcellular distribution of GATA4 mutant protein. The immunofluorescence staining of the transfected HeLa cells shows that the GATA4 p.R311W mutant protein doesn’t change its nuclear distribution.

GATA4 is a nucleic transcription factor and localizes completely in the nucleus [16]. The p.R311W mutation falls within the highly conserved nuclear localization signal domain, a region that is of great importance to protein location. We hypothesized that as a consequence of this mutation, the protein may have altered intracellular distribution, which would prevent GATA4 from properly functioning. To test this hypothesis, we performed immunofluorescence staining. However, the result showed that the wild-type and the mutant GATA4 proteins translocated completely into the nucleus with normal distribution (Fig 4).

### Decreased transcriptional activity of GATA4 mutant protein

**Fig 5:**
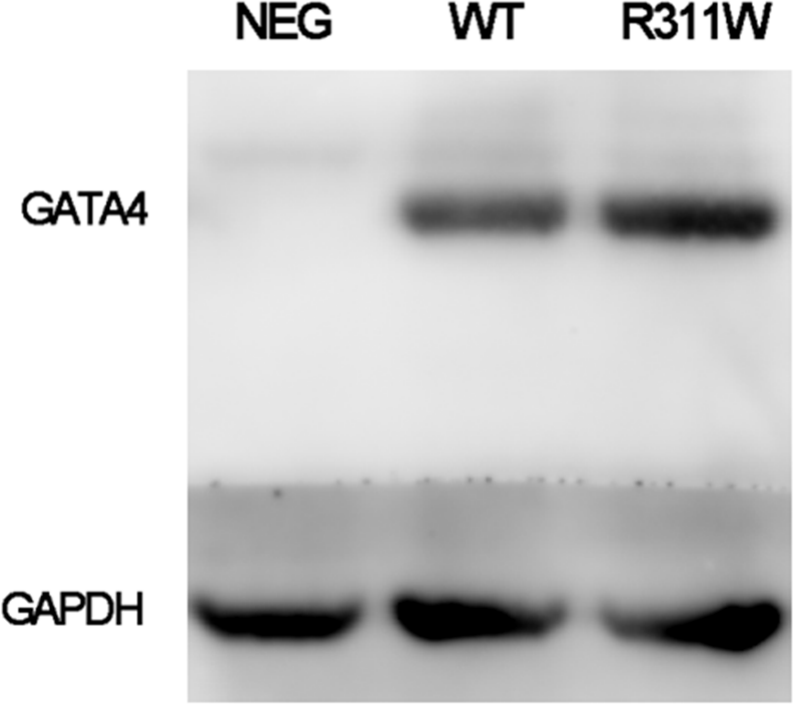
Expression levels of GATA4 wide-type and p.R311W mutant protein in Hela cells. Western blot exhibits equal amount of GATA4 p.R311W protein as compared to the wild-type. Protein loading is normalized by the level of GAPDH.

**Fig 6:**
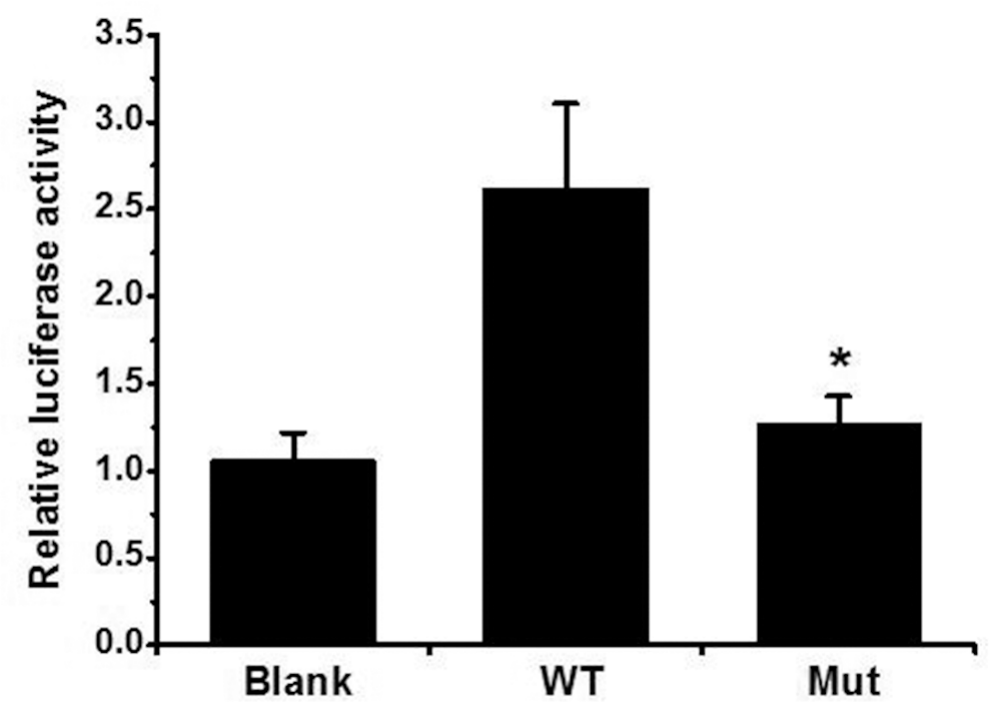
Decreased transcriptional activity of GATA4 mutant protein. Relative luciferase activation of ANF-Luc in Hela cells shows significantly reduced transcriptional activity of the p.R311W mutant protein. (*, P<0.05).

The expression of GATA4 plasmid in Hela cells was quantified by western blot and there was no significant difference between the wide-type and mutant protein (Fig 5, S1 Fig). It has been previously demonstrated that ANF is one of the direct cardiac downstream target genes of GATA4 [17]. To determine whether p.R311W affected the functional activity of GATA4, ANF promoter luciferase reporter together with either wide-type or mutant GATA4 plasmid were co-transfected to HeLa cells. As shown in Fig 6, the p.R311W plasmid present a significantly reduced transactivation of the ANF promoter (P<0.05), demonstrating a diminished target gene activation of the mutant protein.

## Discussion

In this study, we identified a novel heterozygous GATA4 mutation p.R311W. Multiple sequence alignment of GATA4 protein showed that the altered amino acid was highly conserved in mammals. Meanwhile, this mutation was predicted to be damaging or disease causing by several in silico bioinformatic tools (MutationTaster, polyphen2 and SIFT), suggesting that this site p.R311 was vital in the function of GATA4.

As a crucial modulator, GATA4 is required for proper regulation of many genes expressed during heart development, including α-myosin heavy chain, β-myosin heavy chain, brain natriuretic peptide and atrial natriuretic factor (ANF) [9, 18]. GATA4 contains regulatory elements of these genes and can potently activate their promoters. In our research, Dual-Glo luciferase assay was carried out to evaluate the effect of p.R311W on the transactivational function of GATA4 and the result revealed a significantly reduced transcriptional activity on the downstream gene.

Human GATA4 maps to a region of chromosome 8 and consists of 7 exons encoding 442 amino acids. The functional GATA4 protein comprises of two independent transcriptional activation domains (TAD) required for transcriptional activity, two highly conserved zinc finger domains (ZnF) essential for DNA sequence identification and protein-DNA binding, and one nuclear localization signal (NLS) region associated with the subcellular trafficking and distribution of GATA4 [19]. The GATA4 mutation, p.R311W, found in our study is identified in NLS region and may disrupt the normal subcellular localization of GATA4. We performed immunofluorescence staining to determine whether the p.R311 was necessary for the nuclear import of GATA4. The result showed that the mutant GATA4 protein translocated normally into the nucleus. Several mutations in NLS region had been identified in CHD patients (S1 Table) and some of the mutant proteins still accumulated to the nuclei (S2 Fig) [20]. All these findings suggested that not all the mutations in NLS region were sufficient to inhibit GATA4 nuclear targeting. This was consistent with the results revealed by Philips, et al. 2007 [21] that four amino acids Arg283, Arg284, Arg318, Arg320 were essential for GATA4 nuclear localization and proteins with individual mutated site in this region all showed normal nuclear distribution.

Besides the NLS region, p.R311 also locates in the GATA4 DNA-binding domain and can have a dual function in both nuclear import and DNA binding. Even though individual mutation is not sufficient to inhibit the nuclear import, it may impair GATA4 protein-DNA binding [21]. In our study, the amino acid at position 311 of GATA4 changes from arginine to tryptophan. Arginine is ideal for binding negatively charged groups and is inclined to form multiple H-bonds. The opposite charge attraction, the length and flexibility of the side chain, and the ability to produce excellent hydrogen-bonding geometries with nucleobases or phosphate groups make arginine a vital residue for protein-DNA binding [22]. But tryptophan belongs to hydrophobic amino acid carrying aromatic ring. The substitution of strongly basic arginine by neutral tryptophan alters the structure and charge of the residue. The p.R311W mutation may reduce GATA4 protein-DNA binding activity and this is the genetic susceptible basis for the diminished transactivation of downstream target genes.

In our work, we identified a novel heterozygous GATA4 mutation and co-segregated it with CHD in a large pedigree. The affected members in this family had different cardiac phenotypes. It was notable that the proband of this family had TOF, but her mother and other affected members harboring the same mutation had ventricular septal defect, atrial septal defect or patent ductus arteriosus. Congenital heart disease is a multifactorial disorder and both genetic and environment factors have influence on the its occurrence [23]. The genotype-phenotype correlation between gene mutation and CHD is complex. The same CHD subtype can be caused by diverse mutations, and the same mutation may lead to different phenotypes in different patients, even they belong to the same family [16, 17]. The twin studies which provide the most reasonable results for separating genetic factors from environmental effects are good models to investigate the etiology of CHD. In theory, the monozygotic twins have the same genetic background and embryonic development environment, and should have the same phenotype. However, the cardiac defects of them are different in many families [24, 25]. This inconsistency emphasizes the contributions of multiple factors to the pathogenesis of CHD. Complex gene-gene and gene-environment influences can explain why individuals with the same GATA4 mutation have distinct phenotypes in this family.

Treatment of CHDs often involves highly invasive cardiac catheterization and surgery. Close monitoring is needed especially in neonatal life and early childhood of patients. If proper precautionary measures are not taken, these defects will be predisposed to late complications and ultimately cause a decrease in life expectancy [26]. This is frequently life threatening and can cause a profound economic and social burden on patients and their family [27]. The screening of genetic factors associated with CHD is important in clinical practice because it can improve genetic counseling by identifying patients with pathogenic mutations which are related to further cardiac complications and can be transmitted to offspring. Considering that the mortality rate is relatively lower in cases with timely operation, surgical repair of CHD is feasible [28]. The genetic information will be especially useful for this family in our report, because testing for p.R311W in new family members can be translated into effective prevention, accurate diagnosis and efficient treatment [29].

## Conclusions

In this study, we reported a novel GATA4 mutation (p.R311W) in a three generation family with congenital heart disease. The present identification expands the mutation spectrum of GATA4 and provides new clues implicated in the mechanism of CHD.

## Supporting Information

S1 Fig Original western blot result for data in Fig 5. M310V and Q316E are two of the GATA4 mutations located in NLS region which have been identified in CHD patients in other research. Western blot displays equal amount of GATA4 R311W/M310V/Q316E protein as compared to wild-type.
(TIF)

S2 Fig Nuclear Localization of the GATA4 Mutants. The immunofluorescence of the transfected HeLa cells shows that the GATA4 R311W/M310V/Q316E mutant proteins don’t change their nuclear localization.
(TIF)

S1 Table Missense mutations of GATA4 reported in patients with CHD. (PDF)
