# Supplementary figures and images for: A Novel Missense Mutation of GATA4 in a Chinese Family with Congenital Heart Disease
Source: PLoS One. 2016 Jul 8;11(7):e0158904. doi: 10.1371/journal.pone.0158904 (PMC4938561; doi:10.1371/journal.pone.0158904)

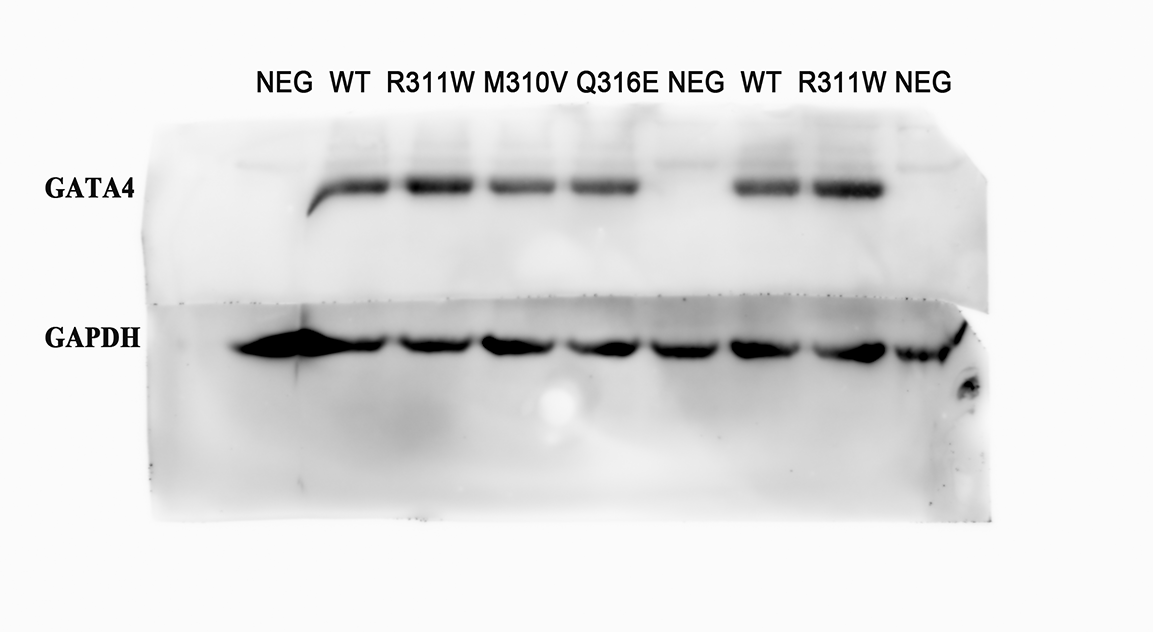

Supplement: S1 Fig — M310V and Q316E are two of the GATA4 mutations located in NLS region which have been identified in CHD patients in other research. Western blot displays equal amount of GATA4 R311W/M310V/Q316E protein as compared to wild-type. (TIF) [file pone.0158904.s001.tif]

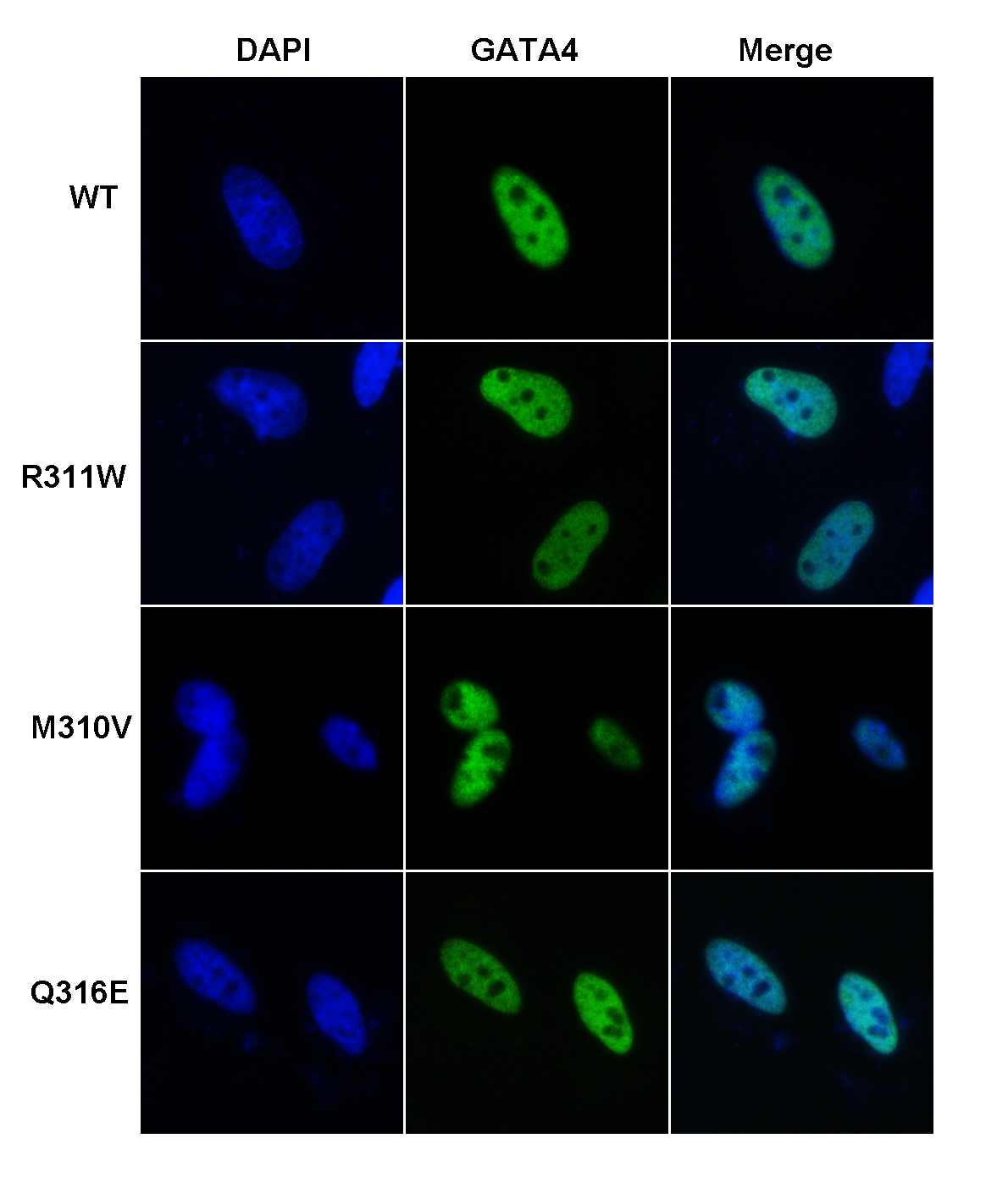

Supplement: S2 Fig — The immunofluorescence of the transfected HeLa cells shows that the GATA4 R311W/M310V/Q316E mutant proteins don’t change their nuclear localization. (TIF) [file pone.0158904.s002.tif]
