# Supplementary material for: A Novel Missense Mutation of GATA4 in a Chinese Family with Congenital Heart Disease
Source: PLoS One. 2016 Jul 8;11(7):e0158904. doi: 10.1371/journal.pone.0158904 (PMC4938561; doi:10.1371/journal.pone.0158904)
Supplement: S1 Table — (PDF) [file pone.0158904.s003.pdf]

No	Nucleotide change	Amino acid change	Region	CHD	Reference
1	c.17 C> T	A6V	TAD1	VSD	Zhang et al., 2008
2	c.25 G>C	A9P	TAD1	TOF	Yang et al., 2013
3	c.46 G>T	G16C	TAD1	AF	Jiang et al., 2011
4	c.62 G>T	G21V	TAD1	ASD	Liu et al., 2011
5	c.82 C>T	H28Y	TAD1	VSD	Chen et al., 2010
6	c.82 C>G	H28D	TAD1	AF	Jiang et al., 2011
7	c.106 C>T	P36S	TAD1	ASD	Yang et al., 2013
8	c.115 G>T	V39L	TAD1	DCM	Li et al., 2014
9	c.127 C>T	R43W	TAD1	VSD	Yang et al., 2012
10	c.151 C>G	L51V	TAD1	TOF	Yang et al., 2013
11	c.155 C>T	S52F	TAD1	ASD	Hirayama-Yamada et al.,2005
12	c.191 G>A	G64E	TAD1	VSD	Yang et al., 2010
13	c.196 G>A	A66T	TAD1	VSD	Chen et al., 2010
	c.196 G>A	A66T	TAD1	VSD/PDA	Wang et al., 2013
14	c.206G>A	G69D	TAD1	ASD	Butler et al., 2010
15	c.209 G>C	S70T	TAD1	AF	Yang et al., 2011
16	c.221 C>A	A74D	TAD1	PS	Wang et al., 2013
17	c.259 C>T	P87S		ASD	Liu et al., 2010
18	c.270 C>T	S90R		CHD	Wang et al., 2010
19	c.278 G>C	G93A		ASD	Tomita-Mitchell et al., 2007
20	c.285 C>G	D95E		CHD	Wang et al., 2010
21	c.448 G>T	G150W	TAD2	TOF	Wang et al., 2013
22	c.470 C>G	S157C	TAD2	VSD	Salazar et al., 2011
23	c.479 G>C	S160T	TAD2	AF	Yang et al., 2011
24	c.487 C > T	P163S	TAD2	VSD/ECD	Zhang et al., 2008
25	c.488C>G	P163R	TAD2	ASD	Butler et al., 2010
26	c.569 A>G	H190R		ASD	Yang et al., 2013
27	c.622 T>C	F208L		VSD	Reamon-Buttner et al., 2005
28	c.628 G>A	D210N		CHD	Wang et al., 2013
29	c.631 T>C	F211L		VSD/AVSD	Reamon-Buttner et al., 2005
30	c.640 G>A	G214S		VSD	Reamon-Buttner et al., 2005
31	c.648 C>G	E216D	ZF1	TOF	Nemer et al., 2006
32	c.661 G>A	G221R	ZF1	DSD and CHD	Lourenço et al., 2011
33	c.668 T>C	M223T	ZF1	VSD	Reamon-Buttner et al., 2005
34	c.677 C>A	P226Q	ZF1	DCM	Li et al., 2014
35	c.687 G>T	R229S	ZF1	VSD	Reamon-Buttner et al., 2005
36	c.700 G>A	G234S	ZF1	AVSD	Reamon-Buttner et al., 2005
37	c.715 A>G	N239D	ZF1	VSD	Reamon-Buttner et al., 2005
38	c.716 A>G	N239S	ZF1	VSD	Reamon-Buttner et al., 2005
39	c.731 A>G	Y244C		VSD	Reamon-Buttner et al., 2005
40	c.740 C>T	M247T		AF	Posch et al.,2010

41	c.743 A>G	N248S		ASD/AVSD	Reamon-Buttner et al., 2005
42	c.749 T>A	I250N		VSD	Wang et al., 2013
43	c.755 G>C	R252P		AVSD	Reamon-Buttner et al., 2005
44	c.764 T>C	I255T		ASD	Reamon-Buttner et al., 2005
45	c.779 G>A	R260Q		VSD	Reamon-Buttner et al., 2005
46	c.782 T>C	L261P		VSD/ASD	Reamon-Buttner et al., 2005
47	c.784 T>G	S262A		ASD	Yang et al., 2013
48	c.788 C>G	A263G		VSD	Xiong et al., 2013
49	c.796 C>T	R266X		ASD, AVSD	Reamon-Buttner et al., 2005
50	c.799 G>A	V267M		ASD	Wang et al., 2010
51	c.818 A>G	N273S	ZF2	AVSD	Reamon-Buttner et al., 2005
52	c.830 C>T	T277I	ZF2	AVSD	Reamon-Buttner et al., 2005
53	c.835 A>T	T279S	ZF2	DCM	Li et al., 2014
54	c.839 C>T	T280M	ZF2	ASD	Chen et al.,2010
55	c.848 G>A	R283H	ZF2	AVSD	Reamon-Buttner et al., 2005
56	c.855 T>C	N285K	ZF2	AVSD	Reamon-Buttner et al., 2005
57	c.854 A>G	N285S	ZF2	TOF	Yang et al., 2013
58	c.871 G>C	V291L	ZF2	DCM	Zhao et al., 2014
59	c.874 T>C	C292R	ZF2	ASD/VSD/AVSD	Reamon-Buttner et al., 2005
60	c.881 C>T	A294V	ZF2	ASD	Reamon-Buttner et al., 2005
61	c.886 G>A	G296S		ASD/VSD	Garg et al., 2003
	c.886 G>A	G296S		ASD	Sarkozy et al., 2005
	c.886 G>A	G296S		ASD	Garg et al., 2003
62	c.886 G>T	G296C		ASD/PS	Rajgopal et al., 2007
63	c.899 A>C	K300T	NLS	ASD	Chen et al., 2016
64	c.905 A>G	H302R	NLS	AVSD	Reamon-Buttner et al., 2005
65	c.928 A>G	M310V	NLS	ASD	Chen et al., 2010
66	c.931 C>T	R311W	NLS	TOF	This report
67	c.946 C>G	Q316E	NLS	ASD	Tomita-Mitchell et al., 2007
68	c.955 A>G	K319E	NLS	ASD/PS	Xiang et al., 2014
69	c.987 C>T	K329N		CHD	Wang et al., 2010
70	c.989 C>G	T330R		PTA	Kodo et al., 2012
71	c.1017 C>A	S339R		PAVSD	Kodo et al., 2012
72	c.1037 C>T	A346V		ECD	Rajgopal et al., 2007
73	c.1057 G>A	A353T		TOF	Wang et al., 2013
74	c.1060 A>G	T354A		ASD	Wang et al., 2010
75	c.1075 G> A	E359K		VSD	Zhang et al., 2008
76	c.1079 A>G	E360G		VSD	Wang et al., 2013
77	c.1081 A>G	M361V		VSD	Reamon-Buettner et al., 2007
78	c.1130 G>A	S377N		VSD	Reamon-Buettner et al., 2007
79	c.1129 A>G	S377G		ASD/VSD/AVSD	Posch et al.,2008
	c.1129 A>G	S377G		CHD	Schluterman et al., 2007
	c.1129 A>G	S377G		VSD/CoA	Salazar et al., 2011

80	c.1138 G>A	V380M		CHD	Schluterman et al., 2007
	c.1138 G>A	V380M		AVSD	Salazar et al., 2011
81	c.1180 C>A	P394T		CHD	Schluterman et al., 2007
82	c.1196 T>G	V399G		VSD	Yang et al., 2013
83	c.1207 C>A	L403M		CHD	Dinesh et al., 2011
84	c.1220 C>A	P407Q		TOF	Zhang et al., 2009
	c.1220 C>A	P407Q		ASD	Wang et al., 2010
	c.1220 C>A	P407Q		TOF	Zhang et al., 2008
85	c.1232 C>T	A411V		VSD	Tomita-Mitchell et al.,2007
	c.1232 C>T	A411V		ASD/PAPVR	Posch et al.,2008
	c.1232 C>T	A411V		AVSD	Butler et al., 2010
86	c.1273 G>A	D425N		ASD/TOF	Tomita-Mitchell et al.,2007
	c.1273 G>A	D425N		VSD	Zhang et al., 2009
87	c.1286 G>C	S429T		VSD	Zhang et al., 2008
88	c.1288 C>G	L430V		ASD	Reamon-Buettner et al., 2007
89	c.1295 T>C	L432S		ASD	Reamon-Buettner et al., 2007
	c.1295 T>C	L432S		CHD	Dinesh et al., 2011
90	c.1306 C>T	H436Y		ASD/VSD/PFO	Chen et al., 2010
91	c.1325 C>T	A442V		VSD	Wang et al., 2013
	c.1325 C>T	A442V		VSD	Zhang et al., 2008
92	c.1324 G>A	A442T		VSD	Reamon-Buettner et al., 2007
